# Perceiving Sound Objects in the *Musique Concrète*

**DOI:** 10.3389/fpsyg.2021.672949

**Published:** 2021-05-20

**Authors:** Rolf Inge Godøy

**Affiliations:** ^1^Department of Musicology, University of Oslo, Oslo, Norway; ^2^RITMO Centre for Interdisciplinary Studies in Rhythm, Time and Motion, University of Oslo, Oslo, Norway

**Keywords:** *musique concrète*, perception, sound object, typology, morphology

## Abstract

In the late 1940s and early 1950s, there emerged a radically new kind of music based on recorded environmental sounds instead of sounds of traditional Western musical instruments. Centered in Paris around the composer, music theorist, engineer, and writer Pierre Schaeffer, this became known as *musique concrète* because of its use of concrete recorded sound fragments, manifesting a departure from the abstract concepts and representations of Western music notation. Furthermore, the term *sound object* was used to denote our perceptual images of such fragments. Sound objects and their features became the focus of an extensive research effort on the perception and cognition of music in general, remarkably anticipating topics of more recent music psychology research. This sound object theory makes extensive use of metaphors, often related to motion shapes, something that can provide holistic representations of perceptually salient, but temporally distributed, features in different kinds of music.

## Introduction

In parallel with the emergence of the *musique concrète*, Pierre Schaeffer and coworkers also dedicated much attention to the perceptual issues of this new kind of music, in particular to the so-called *sound objects* and their various features. Although based on recorded fragments of sound, typically in the 0.5-5 s duration range, sound objects are actually perceptual images of these sound fragments, i.e., images in our minds of sound fragments we listen to. These perceptual images are largely influenced by our individual attitudes and schemas, including our attentional focus during listening. Yet there was also an attempt to find some common features among individual experiences of sound objects by the use of metaphor labels. These labels, largely related to motion shapes such as *impulsive*, *iterative*, *sustained*, *rough*, *smooth*, etc., grew out of practical composition work in the *musique concrète* and ensuing discussions at the *Groupe de recherches musicales* in Paris.

This focus on sound objects and their perceptually salient features was, and still is, a remarkable development in music theory. It is top-down in the sense of taking the subjective (or what we could call experiential) features as point of departure for systematic exploration, all the time guided by the seemingly naïve method of numerous listening to any sound fragment and trying to depict what we are hearing. In being founded on perceived sound, and not on Western notation or other abstract paradigms, this theory emerged ahead of its time, and may from our present day vantage point be regarded as just as much a project of music psychology as of music theory.

The aim of the present paper is first of all to highlight the extraordinary role of the sound object focus in music theory, not only relevant for the *musique concrète*, but relevant well beyond that to many different musical genres and styles, because sound objects are inherently holistic and thus capable of conceptualizing temporally distributed features of music such as the various dynamic, textural, timbral, and expressive envelopes that has not be possible within the more traditional Western music theory frameworks. A follow-up aim here is to demonstrate how the metaphors of Schaeffer and co-workers may be related to the acoustic substrate of sound objects, now that we have readily available methods and technologies to explore such relationships. The empirical material in this paper is then the collection of metaphor labels and their corresponding sound examples in Schaeffer’s theory, as manifest in the text and sound work *Solfège de l*’*objet sonore* (Schaeffer et al., 1998, here called *Solfège*), specifically the labels of the *typology* (the overall dynamic and pitch-related shapes of sound objects), and of the *morphology* (the various detail contents of sound objects).

**FIGURE 1:**
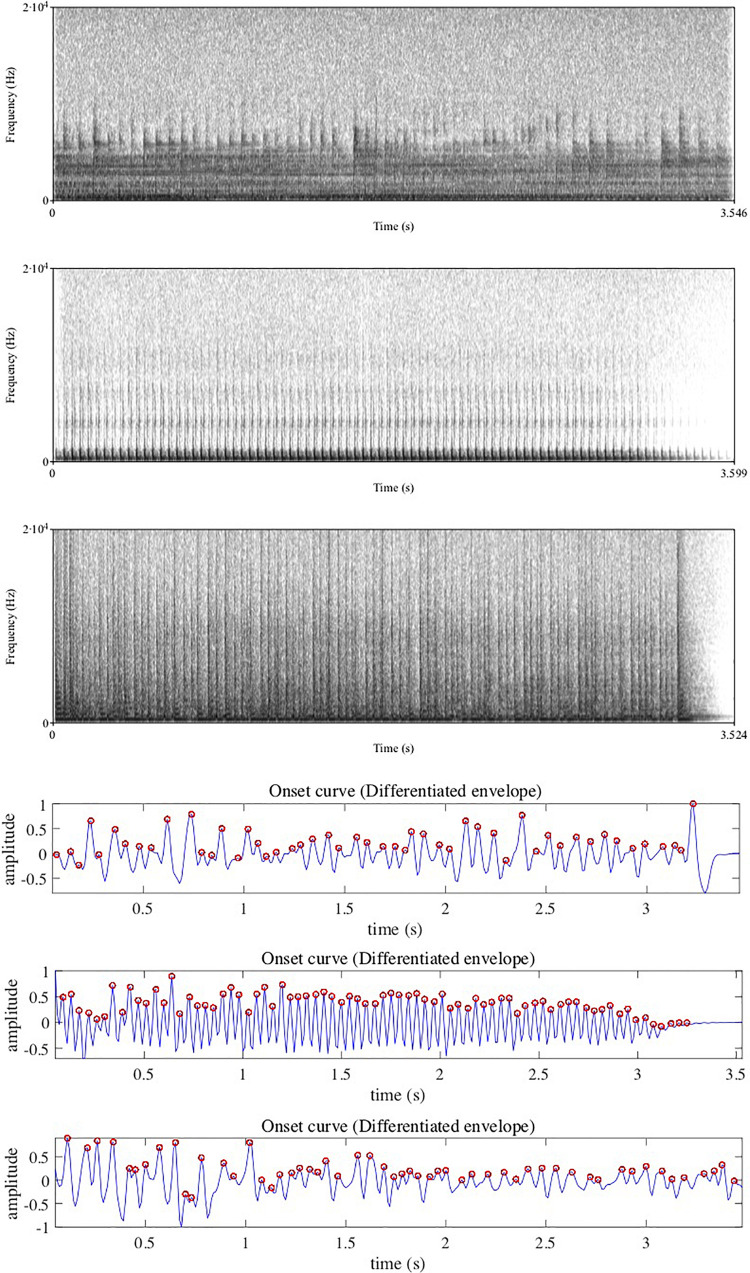
Spectrograms (top part) and MIRtoolbox plotting (bottom part) of amplitude peaks (indicated with small red circles) in the sounds of a drumroll, a deep bassoon tone, and an electronic sound, from *Solfège* CD3, respectively, tracks 25, 26, and 27. These series of amplitude peaks, variably in the region between 15 Hz and 20 Hz, are all perceived as so-called *iterative* textures of the three sound objects in spite of their different origins, illustrating the principle of generic typological features across sounds with different origins. The MIRtoolbox plotting picking out the peaks from the continuous sound signal are based on a derivative function for peak finding in the MIRtoolbox, hence will be quite accurate in locating the peak points in the signal.

The challenge in this paper is to explore how to bridge the gaps between the subjective labels and the acoustic features of the sound objects in research, in particular as these labels are applied across different sounds e.g., across that of a drumroll, a deep bassoon tone, and of a processed sound, all with prominent so-called *iterative* feature (cf. *Solfège* CD3, tracks 25, 26, and 27, see Figure 1 below). The central question is then: is it possible to document the acoustic features of sound objects that constitute the basis for the subjective labels in the *Solfège*? In other words: can we correlate the subjective feature labels of the sound objects with their acoustic features, as was indeed the long-term project of musical research proposed by Schaeffer?

The scheme of this paper is to first present the reasoning for the sound object focus, and then to illustrate the correlation of metaphors and acoustics by going backward from the subjective feature labels to the acoustic features of some sound examples in the *Solfège*, and using available sound analysis tools (such as *Praat* and the *MIRtoolbox*), evaluate how these subjective feature labels actually relate to acoustic features. The hoped for outcome is to point out some correlations between subjective labels and their acoustical substrates in the *Solfège*, and contribute to future research bridging the gaps between subjective labels and acoustic features in music perception studies (be that behavioral or neurocognitive), as well as in practical sound design work.

To better understand this sound object focus, the next two sections will present some background material on the sources and context of the *Musique Concrète*. This will be followed by a presentation of the main challenges of the *Musique Concrète*, its object focus, listening ontologies, and object features, before sections on acoustic correlates, typology, morphology, and multidimensional modeling. Lastly, there will be a discussion of the gains and possible future developments of the sound object theory, in particular in view of readily available technology for analysis and creation.

### Sources of the *Musique Concrète*

Besides being a composer and an engineer, Schaeffer was also a writer with numerous publications (including novels and plays), and he wrote an extensive account of the development, experiments, and thoughts, leading up to the *musique concrète* in his 1952 book, *A la recherche d*’*une musique concrète* Schaeffer, 1952, available in English translation as (Schaeffer, 2012). Part diary, part protocol, and with extensive discussions of the perceptual issues involved, this book is a testimony to Schaeffer’s fascination with the aesthetics of concrete sounds. This book also includes accounts of the composing of early concrete works such as *Etude aux chemins de fer*, using sounds of locomotive engines and horns put together in a kind of score (Schaeffer, 2012, pp. 26-27).

As for the label “musique concrète,” Schaffer wrote: “When in 1948 I suggested the term *musique concrète*, I intended, by this adjective, to express a *reversal* of the way musical work is done. Instead of notating musical ideas using the symbols of music theory, and leaving it to known instruments to realize them, the aim was to gather concrete sound, wherever it came from, and to abstract the musical values it potentially contained.” (Schaeffer, 2017, p. 7). And: “I have coined the term Musique Concrete for this commitment to compose with materials taken from “given” experimental sound in order to emphasize our dependence, no longer on preconceived sound abstractions, but on sound fragments that exist in reality and that are considered as discrete and complete sound objects, even if and above all when they do not fit in with the elementary definitions of music theory.” (Schaeffer, 2012, p. 12).

The terms *concrete* and *abstract* are recurrent in Schaeffer’s writings, and they denote not only the crucial difference between recorded sound and Western music notation symbols, but also the difference between more open-ended concepts of musical sound and the relatively strict categorical schemes of Western notation, i.e., of pitch and duration. In the words of Schaeffer: “The adjective “abstract” is applied to ordinary music because it is initially conceived in the mind, then notated theoretically, and finally executed in an instrumental performance. As for “concrete” music, it is made up of preexisting elements, taken from any sound material, noise, or musical sound, then composed experimentally by direct montage, the result of a series of approximations, which finally gives form to the will to compose contained in rough drafts, without the help of an ordinary musical notation, which becomes impossible.” (ibid, p. 25). It is also interesting that the conceptual apparatus and associated terminology for the sound object focus, was largely developed already in 1952, as can be seen in the section called “Twenty five initial words for a vocabulary,” developed in collaboration with Abraham Moles (Schaeffer, 2012, pp. 194-221).

### Context of the *Musique Concrète*

The concrete-abstract antinomies should also be seen on the background of Western musical modernism in the post-1945 area. Several aesthetic directions existed side by side, but there were some prominent directions that we see allusions to in the writings of Schaeffer. There is the so-called *Darmstadt School* of integral serialism fronted by Boulez, Stockhausen, and Nono (among others), and in spite of its proclamations of radical aesthetic ideas, it was also practicing a conventional Western musical framework of tempered tuning and discrete notation. In the opinions of its critics (including Schaeffer), there was also a lack of focus on the perceptual outcomes of this music. But in a kind of middle position, we may find Xenakis with his schemes of formal and statistical distributions of musical sound, hence partially focused on the emergent sound object shapes and textures (Xenakis, 1992).

In parallel, there was a development of acoustics research, and Schaeffer was evidently quite well versed in this field, both by training as an engineer and by being familiar with mainstream publications in musical acoustics (Helmholtz, Stumpf, as well as Moles, with whom Schaeffer worked). What emerges from Schaeffer’s discussion of acoustics topics is a recognition of the acoustic basis for musical sound combined with reservations about the limitations of acoustic theory in view of experienced sound features. Schaeffer basically claimed there was a non-linear relationship between acoustics and perception, manifest in what he called *anamorphosis*, sometimes rendered in English as “warping,” implying a need for an empirical feature-by-feature mapping between signal and percept. This reservation in Schaeffer’s writings toward relevant insights from acoustics, is something that Michel Chion ascribes to a kind of “scientific” idea in the 1950s and 1960s of the sound itself without taking into consideration the complexities and non-linearities of perception and the listeners intentionality (Chion, 1983, p. 30).

Between the polarities on the one hand, of inherited western notation concepts, and on the other hand, the more physicalist acoustical research, Schaeffer sought to establish a domain of research into perception of musical sound where the sound was considered concrete in the sense of not limited by the abstractions of Western music notation symbols. The core of this research was on sound objects, and as is the main focus of this paper, Schaeffer saw sound objects as an autonomous domain of research based on our subjective experiences, however with a long-term ambition to correlate these sound objects with acoustic features.

The principal source for Schaeffer’s sound object research in this paper is his monumental *Traité des objets musicaux* (Schaeffer, 1966), now fortunately available in English (Schaeffer, 2017). A very useful introduction and overview of this work is in Michel Chion’s *Guide des objects sonores* (Chion, 1983), also available in English (Chion, 2009). The mentioned pedagogical work, *Solfège de l*’*object sonore* (originally published in 1967, but a renewed version containing 3 CDs and a text booklet was made available in Schaeffer et al., 1998), containing sound examples and Schaeffer’s narration, gives an excellent presentation of the main ideas of Schaeffer’s research and will be much referred to here. Schaeffer’s *Traité* (Schaeffer, 2017) may seem dauting in its extension and richness of ideas, and in comparison, the *Solfège* is a simplified yet highly reliable presentation of the main elements of Schaeffer’s theories.

## Challenges of the *Musique Concrète*

Reading Schaeffer’s accounts of the *musique concrète* developments in 1948-1952, we sense a profound fascination with the features of concrete sounds as well as various ideas of how to use these sounds in musical compositions. This included the idea of a kind of “noise piano” akin to Cage’s prepared piano (Schaeffer, 2012, pp. 7-8), yet there was also a need to make some more general kind of structure to all this sound material:

Faced with so many disparate objects, totally without grouping, without their conventions or their natural patrimony, a *classification*, even approximate, is essential, a sort of “grid” completely replacing instrumental tablature or the natural repertoire of noises. For how can we study an infinity of sounds that are not identified in any way? We will therefore use “sound identification criteria.” They will give us the means to isolate sound objects from each other, since we refuse to do this through the usual sound or musical structures. In addition, they will lead us to a practical classification of sound objects, an obvious prerequisite for any further musical regrouping. (Schaeffer, 2017, p. 289).

In (Schaeffer, 2017) there are numerous passages about how to make sense of this disparate material, and with excursions into linguistics, gestalt theory, and phenomenology (to name the most important domains), yet at the same time also recognizing that there was a rather pragmatic beginning to the pervasive focus on sound objects, namely the experience of the so-called *closed groove* (*sillon fermé* in the French original).

The experience of closed groove stems from the use of disks with looped sounds in the *musique concrète* during the years before tape recorders became available. In composition work, this meant mixing sounds from several different phonographs to make the wished for sound texture, but it had the side effect of making people listen to innumerable repetitions of sound fragments. This in turn diverted the listeners’ attention to various features of any sound fragment, first of all to the subjectively perceived overall dynamic shape, or envelope, of the sound fragment, and then, also to the more internal timbral-textural features of the fragment. In the words of Schaeffer:

First, using the closed groove in the early stages of our work with the gramophone (without the closed groove our method would doubtless have never come into being), we made ourselves extract “something” out of the most disparate sound continuum. Thus this surrealist violation, so far removed from the earnestness of our colleagues in electronic music, obliged us to cut up sound and face up to what was most ill-assorted, most resistant to organization. (Schaeffer, 2017, p. 310).

Another and related experience was the so-called *cut bell* (*cloche coupé*e in the French original), denoting the experience of a looped bell sound containing only the resonant part of the sound, i.e., that the attack transient of the mallet stroke had been removed, resulting in a sound that more resembled a sustained flute sound than a bell sound.

The experiences of “closed groove” and “cut bell,” seemingly serendipitous in origin, became decisive for the development of research on sound objects for two main reasons:

(1) It forced the listeners’ attention to the overall shape features of the sound fragment, i.e., to highly salient sound object timescale features that tend to be swallowed up in a longer context of continuous musical sound, e.g., as in that of the timescale of Western musical works, or on the other hand, ignored by the more abstract focus on notated tones.
(2) It forced listeners to accept that sound features may be based on elements spread out in time, and that perception may work on a more holistic basis of taking some segment of musical sound into account as a whole, e.g., that the strike phase, and not only the quasi-stationary phase of the bell sound, is crucial for the perception of is characteristic sound.

### Object Focus

There are other possible approaches to object-formation in auditory perception, and in the literature, we may encounter terms such as *chunking*, *grouping*, *parsing*, often based on qualitative discontinuities such as alternations between sound and silence, shifts in register, changes in spectral features, etc. Yet qualitative discontinuities alone may often be insufficient because of competing cues, missing information, or ambiguities requiring cues in accompanying modalities (See Godøy, 2008 for a summary). In the case of Schaeffer, the approach is first of all based on a top-down subjective perception of the overall shapes of the sound fragment, proceeding downward to more detail shapes, in a process where the closed groove gave the sound object a kind of solid appearance:

The closed groove did, indeed, give an object in the sense of *a thing*, hidden away, as it were, by destroying another object. We have just observed that this involves not so much an objective discovery as putting the participant in a different situation. What does he see now that he had not seen by similarly, breaking up an elementary object, such as the sound of a bell, for example? Breaking it up informs him about the object, which he has—momentarily— destroyed only to hear it better. But if we bring the two experiments together, the closed groove and the cut bell, artificial, strange, anti-musical objects, and if we open our ears, we begin to hear whatever it is, sound or musical, differently, thanks to *reduced listening*, an experience that these two exercises in disruption taught us. (Schaeffer, 2017 p. 311).

The experience of the closed groove and cut bell would then serve to shift our attention as listeners to the overall shape as well as to the more internal features of the sound, away from the usual everyday significations, e.g., away from perceiving the squeaking of a door as a cue that someone is coming, toward the dynamic and spectral features of the squeak. This shift of focus in listening is what Schaeffer called *reduced listening*, with reference to intentionally disregarding everyday significations of sounds in favor of their more intrinsic sound features. But notably so, Schaeffer stated that this shift to focusing on the sound object timescale also was a strategic choice:

We could say, in the most everyday language, that we could tackle the investigation of the musical from both ends—material and works—and *that we have exclusively chosen material*. But to put forward such a clear separation would be to forget the essential connectedness that articulates structures from the simple to the composite and that does not necessarily start with the simple: we enter into such relationships at any level, so we gain access as much to the higher as to the lower levels. In other words we perpetually keep in our minds and ears the part played in every work by *objects* (sound building blocks) that we can isolate and compare with each other independently of the context from which they come. (Schaeffer, 2017, p. 17).

Upon reflection, we may realize that the object focus is not altogether foreign to other music theory. For instance, in music theory texts we encounter the explanation of various clichés, e.g., in the treatment of dissonance (suspension, cambiata, etc.) and ornaments (mordent, trill, etc.) as well as instrumental idioms, as objects, and in this line of thinking, Schaeffer actually lists Messiaen’s birdsongs as objects (Schaeffer, 2012, p. 171). The advantage of having the twin experiences of closed groove and cut bell is then to demonstrate that sound object perception is holistic, and that what occurs sequentially may be perceived holistically.

### Listening Ontologies

Another crucial feature of the concrete music was the use of loudspeakers. With reference to the myth of Pythagoras that he was hiding behind a screen when teaching so that his students should not be distracted by seeing him, Schaeffer adopted the term *acousmatic* to denote the listening situation of concrete music as based on loudspeakers. Having no visual sound-producing source present except for loudspeakers, this meant that all sensations had to come by way of listening. With concrete sound objects in most cases having multiple significations and features, e.g., in terms of everyday significations as well as spectral, dynamic, textural, etc., features, the listening experience will seldom, if ever, be unambiguous, and will depend on the intentional focus of the listener at any time. For this reason, Schaeffer emphasized that listening would produce varying results, yet also suggested that we to a certain extent may control our attentional focus in listening:

Every object perceived through sound is only so because of our listening intention. Nothing can prevent a listener from destabilizing this, going unconsciously from one system to another or else from *reduced* listening to one that *is not*. We should perhaps even congratulate ourselves on this. It is through such swirling intentions that links are established, information exchanged. (Schaeffer, 2017, p. 272).

The general point is that sound sensations can have multiple meanings, i.e., be what we could call *ontologically composite*. And on the way to a comprehensive theory of sound object perception, it could be useful to make an analysis of listening in view of focusing on the musical potential of concrete sounds. To this end, Schaeffer suggested that there are four components in listening (here with the French terms in parentheses for clarity): listening (écouter), perceiving (ouïr), hearing (entendre), and comprehend (comprendre), and that although they in most situations interact, we should distinguish them because they concern different aspects of what we perceive. To summarize the relationship between these four components, Michel Chion made this example sentence: “*I perceived (ouïr) what you said despite myself, although I did not listen (écouter) at the door, but I didn*’*t comprehend (comprendre) what I heard (entendre).*” (Chion, 2009, p. 20).

Furthermore, Schaeffer suggested that listening may proceed by sketches, i.e., that sound object images may develop in our minds by repeated listening experiences: “…the process of *qualified listening*, the diversity of which arises from this fundamental law of perception, which is to proceed “by a series of sketches,” without ever exhausting the object…” (Schaeffer, 2017, p. 77). With reference to Husserl’s ideas on the constitution of objects in our minds by way of multiple but different sensations of the same object (Husserl, 1982), and where the cumulative perceptual image is called the *transcendence* of the object, Schaeffer comments:

And then I notice that it is *in my experience* that this transcendence is *formed:* in other words, the *style* of perception itself, the fact that it never uses up its object, proceeds by rough sketches and always refers to other experiences that may contradict the previous ones and make them appear illusory, is not the sign of an accidental and regrettable imperfection that prevents me from knowing the external world “as it is.” This style is, in fact, the mode in which the world is given to me as distinct from me. It is a particular style that allows me to distinguish the perceived object from the products of my mind or imagination that have other structures of consciousness. *So every domain of objects has its type of “intentionality.” Each of their properties refers back to the activities of the consciousness that are “constitutive” of it: and the perceived object is no longer the cause of my perception. It is its “correlate.”* (Schaeffer, 2017, p. 210).

The point of the correlate is crucial for understanding the relationship between the sound object as a perceptual entity and the acoustic contents of any sound fragment. Exploring this correlation was seen by Schaeffer as the long-term aim of research, whereas his more here-and-now project consisted in mapping out the subjective features of sound objects, notably so that there would be some kind of constancy in the object images in spite of the incessant variations in our images. Under the heading of *Objectivity of the object*, in the *Solfège* CD2, tracks 88 to 95, we can hear a series of examples combining constancy and variation, and also illustrating some of the major sound object feature categories.

### Object Features

In (Schaeffer, 2012, 2017) Schaeffer recounts how he arrived at considering the sound object timescale as the most important in musical experience through some experimental work using (by our present standards) rather simple technologies. The mentioned closed groove and cut bell were the beginning, and this was followed by some other discoveries regarding the role of the attack transients and spectral non-linearities, summarized in the concept of *anamorphosis*, i.e., of warping.

The cut bell experience was considered a *temporal anamorphosis* in the sense of the instrument identity being dependent on sequentially occurring elements. There were also experiences of non-linearities in other domains, such as in the spectral composition of sounds across the range of any single instrument. For instance, a deep piano tone when shifted up a couple of octaves (by increasing the playback speed) sounds more like a harpsichord than a piano, hence, our perception of the unitary “piano-like” sound across the full range of the piano must be due to a more complicated set of factors than a linear shifting of the acoustic signal. This and similar experiences led Schaeffer to suggest that perceptual sound features should be seen as an independent domain, however, related to the acoustic substrates by the mentioned relationship of correlation. This correlation serves to show that the point of departure should be the subjective experience of a sound object and its perceptual features, in other words, that we should proceed in a top-down manner starting with seemingly simple questions as to what we are hearing. Notably so, this top-down feature differentiation may become quite complex, consisting of several main features, sub-features of these main features, and sometimes also sub-sub-features, as can be seen in the *Summary diagram of the theory of musical objects* (Schaeffer, 2017, pp. 464-467).

In view of the possible use of sound objects in musical compositions, Schaeffer introduced the idea of the *suitable object*. The suitable object fulfills some very general criteria, criteria that are flexible and context-dependent, yet interesting here in view of perceptual features. In brief, these criteria may be summarized as:

• The sound object should not be too long, nor too short.
• The sound object should not be too diverse, nor not to uniform.

The most interesting here is probably that or duration, which we can correlate with theories of attention spans found in various phenomenological and/or cognitive science contexts (e.g., in Michon, 1978; Husserl, 1991; Pöppel, 1997) and in recent research on motor control (see Godøy, 2018 for a summary).

As for non-suitable objects, they are either too short or too long, and/or they are either too varied or too unchanging, what Schaeffer denotes as *redundant*. One such sound object is the so-called *large note*, denoting a sound object that in spite of having clear gestalt coherence and closure, is just too long to be a suitable object in future musical compositions (examples of large note in the *Solfège* are up to 30 s). Another is the *ostinato* object that is redundant in its manifold repetitions, and at the other extreme, there are objects that are too short and/or too dense to be called suitable. CD3, tracks 43 to 65 offers examples of these various non-suitable objects.

It may be interesting to compare these durations with some other projects, e.g., that of (Gjerdingen and Perrott, 2008) suggesting that the overall timbral features may be perceived in fragments as short as 250 ms, whereas recognition of various rhythmic and melodic features of course would require longer durations. At the longer end of the timescale, we find object durations typically in the 3 – 15 s duration range, such as the UST (*Unités Sémiotiques Temporelles*) project, partially inspired by Schaeffer’s research, but more focused on semiotic and affective features of sound objects (Delalande et al., 1996).

### Acoustic Correlates of Sound Objects

That Schaeffer characterized the relationship between sound objects and acoustics as that of correlation and not of identity does not mean that there is no basis for sound objects in musical acoustics, but rather that he thought it necessary to make an analysis of factors involved in the perception of sound objects, and then explore the relationship between subjective perceptual sensations and acoustic features. This analysis may include features of what we presently refer to as *psychoacoustics* (as e.g., in Fastl and Zwicker, 2007), but Schaeffer’s theory is broader in scope in that it also includes composition-oriented features, as well as features at the timescale of entire sound objects.

For Schaeffer, the point of departure for this correlation research was the initial subjective sensations of the sound object as a whole, as manifest in the *closed groove* experience, and then proceed to differentiate its various features. The approach is thus top-down, starting with an overall subjective image of any sound object, and progressing downward into the signal-based acoustic substrates.

The first consideration of acoustic correlates is then that of *timescales*, i.e., that acoustic features of sound objects are found at concurrent timescales ranging from the slow of the shape of the entire sound object, i.e., typically in the 0.5-5 s duration range, with overall dynamical, timbral, or pitch-related envelopes, down to the fast oscillations in the range of perceived pitch and/or spectral features, i.e., in the 20-20000 Hz range. A crucial point with the experiences of *closed groove* and *cut bell* was the need to take the entire sound object into consideration, in that all events within the entire sound object, e.g., the strike tone followed by the sustain tone and the ensuing overall dynamical, spectral, pitch-related, etc., envelopes of the entire sound object, contribute to its perceptual image. This requires that the entire sound object be kept in echoic memory (Snyder, 2000), so that sequentially unfolding elements may be present in our minds “all-at-once.”

The second consideration of acoustic correlates is that sound objects may be situated in a *multidimensional model* consisting of main features, sub-features, sub-sub-features, as well as various values for these different feature dimensions, i.e., scales between minimum and maximum values, e.g., the feature of tremolo (amplitude modulation) may occur at a range between a maximum and a minimum speed. And with the possibility of intentional focus in listening, we may also zoom in and out of features at different timescales, i.e., from the overall shape of the sound object to its most minute transients, something that Schaeffer denoted as the “two infinities” of sound objects (Schaeffer, 2017, p. 220). A timescale analysis is then crucial for understanding the differentiation of acoustic features in Schaeffer’s theory, as reflected in these three main categories:

• The *typology* timescale, with various sub-categories, can encompass features in the duration range from that of the entire sound object down to patterns within a sound object, typically concerning overall dynamic and spectral shapes of sound objects, providing a coarse sorting of sound objects.
• The *morphology* timescale, with various sub-categories, typically including more internal features of the sound objects such as its pitch, timbre, spectral shapes, as well as various rapid internal fluctuations and transients.
• The combination of all main features in the multidimensional model, rendered in English as *“Summary diagram of the theory of musical objects”* (Schaeffer, 2017, and pp. 464-467), which presents an overview of the various typological and morphological main dimensions and sub-dimensions, as well as relative values for these dimensions, e.g., the sound object of a rapid harp glissando combining an overall dynamic and pitch-related shape with detail shapes of individual tone onsets and timbres.

The idea of such multidimensional modeling of musical sound has later been developed in the works of e.g., John Gray, David Wessel, and others, and with implementations in software such as e.g., in the *Timbre Toolbox* (Peeters et al., 2011) as well as in some projects within so-called *music information retrieval*, e.g., the *MIRtoolbox* (Lartillot and Toiviainen, 2007). Besides being remarkably ahead of his time, Schaeffer’s multidimensional model is also striking in its generality, making it applicable to any kind of music (e.g., *musique concrète*, various avantgarde or various non-Western music). Another advantage is the strong top-down direction of detecting and qualifying perceptually salient features, rather than a more “blind” and/or purely signal-based, bottom-up search for significant features.

Guided by subjective perceptual categories, this multidimensional model can relate to most (or all) traditional musical acoustic elements such as pitch, stationary spectra, various time domain and frequency domain envelopes, but with the additional advantage of naming salient features that are based on more composite acoustic features such as that of *gait* and *grain* in the morphology (see section “Morphology” below). This is in particular useful for capturing components of timbre and musical textures that rely on transients and fluctuations, hence are not limited to stationary spectra.

This multidimensional scheme can distinguish different values for salient components, e.g., the rate and amplitude of fluctuations within a sound objects. Changes in rates and amplitude may then help us distinguish different categorical thresholds for salient features, e.g., that of the rate and amplitude of a frequency modulation: if the rate is slow (say no more than 8 Hz) we may perceive a vibrato, but if it is significantly faster (say above 20 Hz) it will become a timbral feature. Similar value thresholds may be found in most (or all) other features and can also be explored by an *analysis-by-synthesis* approach (Risset, 1991), similar to what we may hear on CD2, track 89 and onward. Categorical threshold explorations may also be found at the object shape timescale, such as on CD3, track 60-63, with an incremental change from a protracted ostinato sound object to a series of singular impulse objects.

The key to exploring the acoustic correlates of sound object is then the two-step process of (a) distinguishing and naming some perceptually salient feature of the sound objects, e.g., its fluctuation in amplitude, and then (b) qualify its value, i.e., its rate, shape, regularity, etc., of fluctuation. We could also add a third step, (c) if we have the means to do so, to generate variants of this feature and evaluate the result, i.e., make incrementally different sound objects by varying the rate, amplitude, shape, regularity, etc., hence, engage in a process of analysis-by-synthesis to explore various categorical thresholds. In brief, Schaeffer’s multidimensional view of acoustics is a remarkable project of making a large number of previously non-thematized but highly salient perceptual features accessible for intentional focus in research and in artistic creation.

Throughout these considerations of salient perceptual features of sound objects and their acoustic correlates, there is a pervasive use of shape-related metaphors, in particular for time domain features such as dynamic envelopes, as well as for spectral elements. This penchant for what we could call “shape cognition” was present already in Schaeffer’s early work on the *musique concrète* in a number of graphical images (Schaeffer, 2012), in particular concerning the attack shapes of sound objects (see e.g., Schaeffer, 2012, p. 203) and is a further testimony to perceptual features as distributed and holistic, i.e., not reducible to more abstract symbolic representation as has been the dominant trend in Western music theory.

### Typology

The typology has two basic dimensions, one concerned with the dynamic shapes, i.e., what we could call the energy envelopes of the sound objects, and what Schaeffer called *facture* types, and one concerned with the spectral features of the sound objects, what Schaeffer called *mass* types, be that as clear pitch sensations or as more ambiguous inharmonic and/or noise-dominated sensations. The three main facture types are the following:

• *Impulsive*, a fast and short sound, typically as by striking or plucking.
• *Sustained*, a prolonged sound with a steady level energy envelope.
• *Iterative*, fast back-and-forth or rotational motion, resulting in a stream of impulses as can be seen in Figure 1 where the sounds of a drumroll, a deep bassoon tone, and a processed sound are compared in view of the common feature of a pronounced iterative feature.

The concept of facture refers to how things are made, so the mentioned three categories can also be related to similar categories of sound-producing motions. This means that an impulsive motion is fast and brief (sometimes also called *ballistic*), and a sustained motion is a protracted and smooth motion, whereas the iterative motion typically is a back-and-forth shaking or rotating motion. The link between the sound facture and motion categories is interesting also in that motion categories are mutually exclusive (e.g., motion can’t be sustained and impulsive at the same time) and refer to basic biomechanical and motor control constraints. Furthermore, there is the link with what may be called the *motormimetic* element in perception and cognition (Godøy, 2003), suggesting that we may also make a multimodal view of these categories in Schaeffer’s theory (Godøy, 2006).

In terms of pitch and/or spectrum, the typology has three main mass types:

• *Tonic*, meaning a perceivable and stable pitch.
• *Complex*, denoting inharmonic or noise-dominated sound.
• *Varied*, encompassing any sound that changes in pitch or frequency placement.

All these categories are well illustrated with examples in the *Solfège* CD3, tracks 28, 29, 30 with respectively, a tonic mass, complex mass, and varied mass.

The typology is a coarse but flexible and universally applicable categorical scheme in which the facture and mass categories can also be combined in a 3 × 3 matrix as follows, first with traditional musical instruments in CD3, tracks 31, 32, and 33:

• Track 31: tonic impulsive, tonic sustained, tonic iterative.
• Track 32: impulsive complex mass, complex sustained mass, iterative complex mass.
• Track 33: impulsive varied mass, sustained varied mass, iterative varied mass.

**FIGURE 2:**
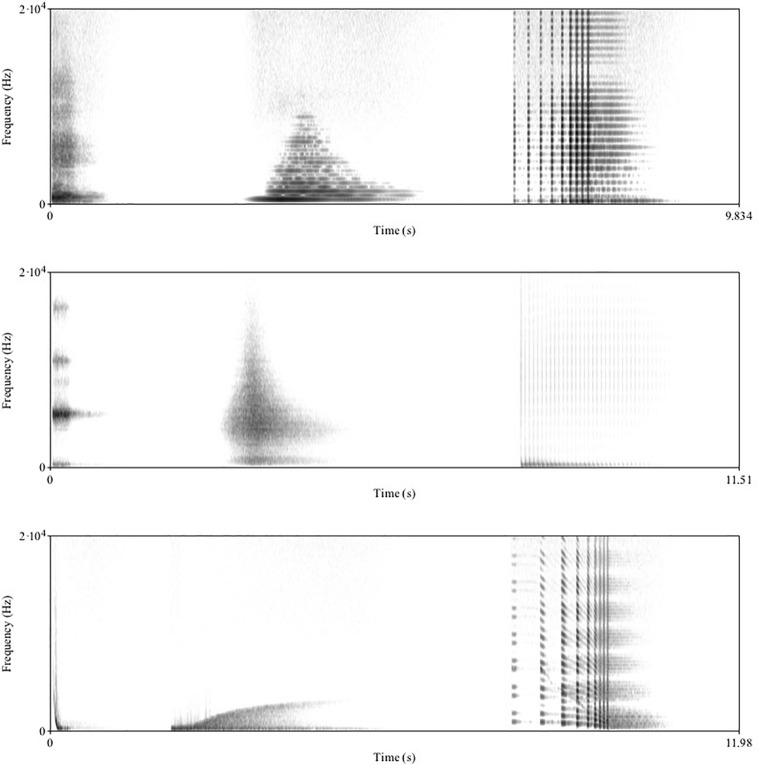
A 3 × 3 typological matrix of spectrograms based on electronic sounds from *Solfège* CD3, tracks 40, 41, and 42. In the first row, spectrograms of track 40 with tonic mass, i.e., clearly perceivable pitches, in the second row, spectrograms of track 41 with complex mass, i.e. inharmonic and noise-dominated sounds, and in the third row, track 42 spectrograms with varied mass, i.e., glissando pitches. In each row, the envelopes have respectively, an impulsive, a sustained, and an iterative facture (see main text for explanation of “facture”). Notice the overall dynamic envelopes of these different factures, i.e., the abrupt of the impulsive, the prolonged of the sustained, and the punctual and accelerating of the iterative.

Then follows a similar scheme with prepared piano sounds repeated in tracks 34, 35, and 36, and with concrete sounds repeated in tracks 37, 38, and 39, as well as with electronic sounds repeated in track 40, 41, and 42. In Figure 2, we can see spectral representations in the 3 × 3 typological matrix based on the electronic sounds of tracks 40, 41, and 42. The overall facture shapes of these 9 different sounds should be quite clear, in spite of different mass content.

### Morphology

The morphology is mainly concerned with the internal features of sound objects, and has several dimensions and sub-dimensions, and for some features, also relative values, e.g., indicating the speed and amplitude of various fluctuations. The morphology is a rather extensive domain, here illustrated with only two of its dimensions:

• *Grain* designates the fast and small fluctuations in the sound, and has attributes such as variations in amplitude, speed, and consistency.
• *Gait* denotes the slower fluctuations in the sound, similar in pace with walking or dancing, but may have variable speed, amplitude, and quality such as mechanical, living, natural, orderly, disorderly, etc.

Additionally, there are morphology dimensions such as *mass* (denoting the overall spectral content, cf. the definition above in the context of the typology), *dynamics* (for the overall loudness), *harmonic timbre* (spectral distribution), *melodic profile* (pitch-related shapes), and *profile of mass* (spectral shapes). This means that the morphology contains both features that are stationary (or quasi stationary) and changing, be that as rapid fluctuations of the grain or more slowly of the gait. Crucially, the morphology features are eminently top-down with sub-dimensions, sub-sub-dimensions, etc., where the guiding principle of exploration is the increasing distinguishing of detail features.

Lastly, there is the possibility of so-called *phase transitions* between various typological and morphological categories. Depending on the density and duration of events, there may be a shift from one category to another, for instance, if an iterative sound is speeded up, it may turn into a grain feature, and conversely, if it is slowed down, it may turn into a series of impulsive sounds. Or: if an impulsive sound is prolonged beyond a certain limit, it may turn into a sustained sound, and conversely, a sustained sound shortened beyond a certain limit will turn into an impulsive sound. The main point here is that we have categorical thresholds where features have more or less typical value ranges, value ranges that may very well be rooted in various perceptual-cognitive schemata of our organism.

**FIGURE 3:**
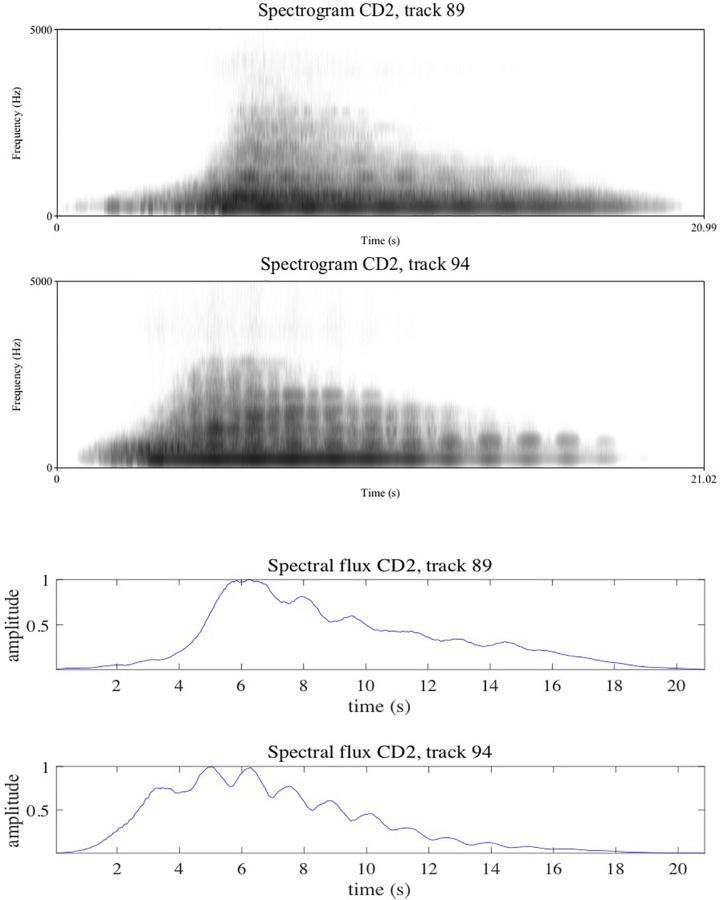
The gait (*allure* in the French original) from *Solfège* CD2, track 89 in the first row and track 94 in the second row, with the gait exaggerated in this second row, both shown here as spectrograms and MIRtoolbox plotting of spectral flux, i.e., plotting of changes in spectral spread, reflecting the wavy motion of the gait. The sound in track 89 is of a tremolo crescendo on a tam-tam followed by a long decay tail with the wavy gait motion at around 2 Hz, and in track 94, the same sequence of events, however, here the amplitude of the wavy gait is strongly exaggerated.

As an illustration of some of these different typological and morphological features, and linked with what Schaeffer called *Objectivity of the object* in track 88 of the *Solfège* CD2, it is instructive to consider the perception of invariance across the series of variants of a sound object starting with track 89: in track 90, a variant of its overall shape, in track 91, a variant of its mass, in track 92, a variant of its grain, in track 93, an exaggerated harmonic timbre, in track 94, an exaggerated gait, and then all combined in the resultant exaggerated object in track 95. This was meant by Schaeffer as an illustration of what a series of listening intentions on the one and same object may bring about, but here manifest in the acoustic correlates of the sound object. In Figure 3, we can see the spectrum and spectral flux (changes in the spectral width) of track 89 into the exaggerated gait in track 94.

### Summary Diagram of the Theory of Musical Objects

Toward the end of CD3 of the *Solfège*, from track 64 and onward, Schaeffer’s conceptual apparatus is put into practice, with an anecdotic account of a composition factory where masses of sound arrive by truckloads, is processed further, and then put together. In these tracks of CD3, we can hear how electronic (both concrete and synthetic) and instrumental sounds, can all be handled with the same perceptual categorical apparatus.

We have an overview of this perceptual categorical apparatus in the mentioned *Summary diagram of the theory of musical objects* (Schaeffer, 2017, pp. 464-467). There we see the main dimensions, sub-dimensions, sub-sub-dimensions, and relative values for these dimensions, enabling a positioning of any sound object in a multidimensional feature model. And this is the take home message from Schaeffer’s theory: we may finetune our perceptual images of sound objects, both in the *musique concrète* and other music, and also apply this conceptual scheme to sound design or composition as part of musical craftsmanship.

Michel Chion reminds us that this diagram is a tool for investigation, that “The general procedure in this music theory is to move forward in a series of approximations rather than in a straight line.” (Chion, 2009, 100). This back-and-forth of sound object and its typological and morphological features, resembling a kind of hermeneutic circle, is one of the key features of exploring sound objects: detecting and naming features enhance our awareness, and this awareness makes us in turn detect more detail features, progressively building up richer and more many-facetted images of sound objects.

## Discussion: Exploring Sound Objects Beyond the *Musique Concrète*

*Musique concrète* was a remarkable project of aesthetic innovation combined with reflections on perception, and in this respect quite different from other contemporary music (cf. section “Listening Ontologies” above). This intrinsic focus on perceptual issues seems to have been driven by the more or less total lack of conceptual tools in mainstream Western music theory for new sounds and sound features, encouraging Schaeffer and co-workers to think outside the box on what were (and are) fundamental questions of sound features and aesthetic judgment (cf. the mentioned ideas about the *suitable object*).

The main elements of Schaeffer’s and co-workers strategy for developing a new and more comprehensive music theory that also included the perception of concrete sound, can be summarized in the concept of the sound object and its typological and morphological features. As found in the *Summary diagram of the theory of musical objects* mentioned above, the most striking elements of this typology and morphology are:

• The multidimensional scheme of detecting, and further differentiating, what are considered salient features of any sound object.
• The all-pervasive element of what we can call *shape cognition* (Godøy, 2019). This concerns not only the verbal labels and graphic signs in the *Summary diagram of the theory of musical objects*, but also the numerous graphical figures in the course of Schaeffer’s work, representing the various attack and spectral components of the sound objects (e.g., shape images for attacks in Schaeffer, 2017, p. 425, and spectral components in Schaeffer, 2012, p. 210).

As for the sound object focus, it is about having holistic perceptions of temporally unfolding fragments of sound. That there always is this holistic element at work for sound objects is reflected by Schaeffer in the example of cutting an object into smaller parts: each new part has a head, body, and tail, just like a magnet cut into two parts will immediately have polarities in each new part (Schaeffer et al., 1998). In this way, any arbitrary cut in a continuous sound recording will result in new sound objects, albeit in the case of a totally random cut, the resultant sound object may not be particularly useful in a musical context, cf. the abovementioned criteria for the so-called suitable object.

It is the overall energy envelope of the sound object that usually will be most prominent, cf. the mentioned typological facture categories, and as such, may be linked with various criteria for chunk-formation in the cognitive sciences, ranging from the classics of Lashley (1951); Miller (1956) to the more recent of Gobet et al. (2016), as well as some ecologically oriented schemes for auditory chunk formation (e.g., Bregman, 1990; Gaver, 1993; Bizley and Cohen, 2013, see Godøy, 2018 for overviews). Importantly, there are also links to chunking in sound-producing motion (Godøy, 2013, 2014), in turn related to chunk formation in body motion (Grafton and Hamilton, 2007; Klapp and Jagacinski, 2011; Loram et al., 2011).

Considering the crucial role of the shapes of sound objects and their features as depicted in the typology and morphology, implies that shape cognition goes right to the core of *musique concrète*. In having moved outside the Western note-symbol domain, we arrive at a more general and sound-centered domain where we are concerned with temporally (and usually also spectrally) distributed, non-abstract entities. This also means we open the door to many traditionally non-thematized features in Western music, in particular concerning timbre, but also various expressive fluctuations of intonation, dynamics, and tempo, with the common feature of not being reducible to singular symbols, but actually requiring some kind of shape representation.

Several projects within the cognitive sciences converge in regarding shape images as fundamental for human perception and cognition (see Godøy, 2019 for an overview). In particular, the so-called *morphodynamical* domain has contributed strongly to recognizing shape images as crucial in understanding and handling complex sensory streams (Thom, 1983; Petitot, 1990; Godøy, 1997). In the words of René Thom: “…the first objective is to characterize a phenomenon as shape, as a “spatial” shape. To understand means first of all to geometrise.” (Thom, 1983, p. 6).

From the various sound examples in the *Solfège*, it is clear that there was an affinity of electroacoustic and ordinary acoustic music in the mind of Schaffer: the typology, morphology, and associated concepts are equally applicable to all kinds of music, i.e., just as well applicable to instrumental, vocal, orchestral, etc., music, as to electroacoustic music. In particular, the typological and morphological categories could be relevant in the analysis of orchestration (see Godøy, 2018 for some examples).

As for sources of shape representations, we have the signal-based, i.e., time-domain and frequency-domain, images. These may be subject to further levels of processing and schemes of representation, selectively representing a variety of perceptually salient features as suggested by the typology and morphology of sound objects. Additionally, there are also the connection to motor theory images of sound, with similar schemes of shape cognition (Godøy, 2003, 2006, 2010), providing multimodal links using motion data in synchrony with sound features data.

Lastly, these shape-oriented explorations of perceptual features can be linked with large-scale perceptual surveys/experiments using music information retrieval software such as the *MIRtoolbox* (Lartillot and Toiviainen, 2007) for exploring correlations between sound objects feature shapes and acoustic signal features, as was the long-term project of Schaeffer.

## Author Contributions

The author confirms being the sole contributor of this work and has approved it for publication.

## Conflict of Interest

The author declares that the research was conducted in the absence of any commercial or financial relationships that could be construed as a potential conflict of interest.
